# Broiler Age Differently Affects Apparent Metabolizable Energy and Net Energy of Expanded Soybean Meal

**DOI:** 10.3390/ani14081198

**Published:** 2024-04-16

**Authors:** Qiuyu Jiang, Yongfa Liu, Zhibin Ban, Bingkun Zhang

**Affiliations:** 1 State Key Laboratory of Animal Nutrition, College of Animal Science and Technology, China Agricultural University, No. 2, Yuanmingyuan West Road, Haidian District, Beijing 100193, China; jqycau@163.com (Q.J.); dklyf@cau.edu.cn (Y.L.); banzb0620@163.com (Z.B.); 2 Laboratory of Animal Nutrition Metabolism, Jilin Academy of Agricultural Sciences, Gongzhuling 136100, China

**Keywords:** energy value, age, heat production, difference method, indirect calorimetric method

## Abstract

**Simple Summary:**

Expanded soybean meal has been widely used in the poultry industry. Precisely evaluating the available energy value of expanded soybean meal by using the difference method will provide important information for feed formulation. Replacing the energy-yielding ingredients with 25% expanded soybean meal by using the difference method increased nitrogen intake, excreta, and energy deposition as protein of broilers. It was found that bird age had remarkable effects on the nitrogen-corrected apparent metabolizable energy (AMEn) and efficiency of net energy to AME (NE/AME) of expanded soybean meal. The results are expected to provide reliable and available energy values of expanded soybean meals and optimize feed formulations for broilers at different ages.

**Abstract:**

Accurately determining the energy values of ingredients is crucial for meeting energy requirements and achieving maximum production performance of animals. This study was conducted to measure the available energy values of three expanded soybean meals (**ESBMs**) for Arbor Acres male broilers from 14 to 16 day and 28 to 30 day using the difference method. A corn–soybean basal diet was formulated, and test diets were developed with 25% ESBMs as substitutes for energy-yielding ingredients. A completely randomized design was used for determining heat production and energy balance of broilers in 12 open-circuit respiration chambers, with six replicates per group. Prior to measurement, four (14 to 16 day) or two (28 to 30 day) birds per chamber were given a 4-day adaption to diets and chambers. The period lasted for 3 days to determine the apparent metabolizable energy (**AME**), nitrogen balance, gas exchanges, and heat production. Broilers fed test diets with 25% ESBM exhibited higher nitrogen intake (*p* < 0.05), nitrogen excreta (*p* < 0.05), and increased energy deposition as protein irrespective of age (*p* < 0.05). Furthermore, results showed that AME, nitrogen corrected AME (**AMEn**), and net energy (**NE**) values of 3 ESBMs averaged 10.48, 8.93, and 6.88 MJ/kg for broilers from 14 to 16 day, while averaged 11.91, 10.42, and 6.43 MJ/kg for broilers from 28 to 30 day. Broilers from 28 to 30 day showed significantly higher AMEn values but lower NE/AME values of ESBMs compared with those from 14 to 16 day (*p* < 0.05). Therefore, age-dependent energy values of a single ingredient should be considered in feed formulations to optimize economic returns.

## 1. Introduction

Soybean meal (**SBM**), with a well-balanced amino acid profile, is the primary protein feed used in the poultry industry. The available energy of SBM for broilers has been measured in previous studies [1,2,3]. In recent years, expanded soybean meal (**ESBM**) has been extensively used in the poultry industry. The expansion technology modified the physicochemical properties of SBM and inactivated anti-nutritional factors [4]. Replacing SBM with ESBM increased nutrients and energy digestibility [5] and improved growth performance of broilers [6]. Hence, precisely determining the energy levels of ESBM for broilers could promote the application of ESBM in the poultry industry.

The metabolizable energy (**ME**) system is widely used in poultry, mainly because feces and urinary losses of birds are voided together through the cloaca. However, the net energy (**NE**) system provides a more accurate estimate of dietary energy and represents the actual energy demand for maintenance and production [7]. NE values consider heat increment (HI) produced during the digestion and absorption process in the gastrointestinal tract [8]. The heat increment of protein is higher than that of fat and carbohydrate in animals [9]. Hence, the application of the NE system potentially reduces the addition ratio of protein feed and decreases nitrogen emissions, resulting in cost savings without detrimental effects on the performance parameters of animals [10,11]. Therefore, it is vital to measure the net energy of ingredients and promote the development of the NE system for broilers.

Factors associated with birds, including strain, age, and body weight, could affect the energy utilization of feeds [12,13]. As broilers grew older, the secretion of digestive enzymes, intestinal surface area, and digestive capacity, especially for SBM, increased [14]. It is easy to illustrate that energy retained in the body improves with increasing age of broilers [15]. AME values of feed ingredients, including corn, soybean, and bran, were reported to be higher in older broilers than in younger ones [16,17]. However, the age-dependent pattern of net energy values of ingredients for broilers is still unknown. A previous study reported that age affects HI due to changes in feed intake and intestinal digestive status of pigs [18]. It is reasonable to speculate that the NE values of ingredients differ between birds at different ages. The objective of the study was to determine ME and NE values of ESBMs for broilers from 14 to 16 day and 28 to 30 day using the difference method. The results of this study could facilitate precise feed formulation.

## 2. Materials and Methods

The experiment was approved by the Institutional Animal Care and Use Committee of China Agricultural University (Beijing, China) for scientific purposes (AW51304202).

### 2.1. Expanded Soybean Meal and Diets

The ESBMs with different crude protein (**CP**) contents were acquired from commercial plants (Wellhope Co., Ltd., Chengdu, China, New Hope Co., Ltd., Shenyang, China and Techlex Co., Ltd., Zhuozhou, China). The measured nutrient contents of three ESBM samples are shown in Table 1. The basal diets were formulated to meet the energy and nutrient requirements for broilers in the grower and finisher phase (Table 2). A corn–soybean meal basal diet was formulated, and three test diets, each containing an ESBM sample, were developed by replacing 25% of the energy-yielding ingredients in basal diets, including corn, soybean meal, corn gluten meal, distillers dried grains with solubles, peanut meal and amino acids (Table 3). 

### 2.2. Birds and Experiment Design

A total of ninety-six 10-day-old and forty-eight 24-day-old Arbor Acres broiler chickens with average body weight (**BW**) were sourced from Tieling city (Liaoning province, China) and housed in a climate-control shed at Jilin Academy of Agricultural Sciences (Gongzhuling, Jilin province, China). Broilers were given ad libitum access to feed and water. After dietary adaption, broilers were transferred from the shed to respiration chambers for 2 days of chamber adaption. A randomized design was used to evaluate four different diets in 12 calorimetry chambers (3 chambers per treatment) for 3 days. Each chamber represents a repeat run. The experiment was conducted in two batches, with each diet repeating six runs, with four (14 to 16 day) or two (28 to 30 day) birds per repeat. 

### 2.3. Respiration Chambers 

Twelve open-circuit respiration chambers (90 × 60 × 100 cm^3^ glass box with an automatic door on the top side) were used in our study. Briefly, chambers were equipped with a vacuum pump, as well as CO_2_ and O_2_ sensors. A zirconium oxide sensor (Model 65-4-20; Advanced Micro Instruments Inc., Huntington Beach, CA, USA) was used for O_2_ detection, and a non-dispersive infrared sensor (AGM 10; Sensors Europe GmbH, Erkrath, Germany) was used for CO_2_ detection. The real-time data of CO_2_ production and O_2_ consumption were collected at 3 min intervals and were expressed as L/min. The respiratory quotient (RQ) was automatically calculated. Moreover, the respiratory chamber was air-conditioned to maintain constant temperature (27 to 30 °C and 23 to 26 °C for broilers from 10 to 16 day and 24 to 30 day, respectively) and humidity (50% to 70%). The analyzer measured a range of 0% to 25% of O_2_ and 0% to 2.5% of CO_2_. In addition, it was suspended for about 1 h each day to replenish feed and collect excreta.

### 2.4. AME Measurement and Chemical Analysis

The AME values were measured by using the total collection method as described by Tillman and Waldroup [19]. Feed intake (**FI**) was measured and calculated daily. Excreta was collected every morning of the testing period. Then, the excreta of six replicates were mixed, oven-dried, weighted, and grounded until through a 1 mm^2^ screen. Feed and excreta were analyzed on a dry matter basis. Gross energy (**GE**) was determined using a bomb calorimeter (IKA-C3000, Bitterfeld-Wolfen, Germany). The CP was determined by using the Kjeldahl method (AOAC, 984.13) with Foss KT200 (Hilloerod, Denmark) [20]. The neutral detergent fiber (**NDF**) and acid detergent fiber (**ADF**) were determined using an Ankom220 Fiber Analyzer (Ankom Technology, NY, USA) with filter bags as described by Van Soest et al. [21]. AOAC methods were also used for the measurement of ether extract [22] and ash (942.05) [20]. The contents of sucrose, stachyose, and raffinose in ESBM samples were determined by using high-performance liquid chromatography (1260, Agilent Technologies Inc., Santa Clara, CA, USA) according to Kennedy et al. [23]. Amino acids were determined using the acid hydrolysis method with an automatic Amino Acid Analyzer (ARACUS, Membrapure, Berlin, Germany). Briefly, the ESBM samples were hydrolyzed with 6 M HCl at 110 °C for 24 h. The samples were then equalized to a 50 mL volume, deacidified, dissolved in sample buffer, and analyzed.

### 2.5. Calculation 

#### 2.5.1. Respiratory Data 

All respiratory data were corrected by BW^0.70^ (Table S1). The heat production (**HP**) was calculated daily following the equation first proposed by Brouwer [24].

HP (kJ/kg BW^0.70^/day) =16.18 × VO_2_(L/kg BW^0.70^/day) + 5.02 × VCO_2_(L/kg BW^0.70^/day)

The respiratory quotient (**RQ**) corresponds to the ratio between CO_2_ production and O_2_ consumption. 

The fasting heat production (**FHP**) value of 450 kJ/kg BW^0.70^/day used in this study was measured by Noblet et al. [25].

HI (kJ/kg BW^0.70^/day) = HP − FHP

#### 2.5.2. Energy Values of Diets

AME (MJ/kg DM) = (GE intake − GE excreta)/FI

AME was corrected to zero nitrogen retention (**AMEn**) using 34.41 kJ/g of nitrogen [26].

NE (MJ/kg DM) = (AME intake − HI)/FI

AME intake was calculated as AME multiplied by FI. 

#### 2.5.3. Retained Nitrogen and Retained Energy

Retained nitrogen (**RN**, g/day) = nitrogen intake − nitrogen excreta

Retained energy (**RE**, kJ/kg BW^0.70^/day) = AME intake − HP [2].

RE as protein (kJ/kg BW^0.70^/day) was calculated as RN × 6.25 × 23.86, according to the previous study [27], while RE as fat was calculated by subtracting RE as protein from total RE. 

#### 2.5.4. Energy Values of ESBMs

Energy values (GE, AME, AMEn or NE) of ESBM (MJ/kg) = (test diet GE, AME, AMEn or NE—basal diet GE, AME, AMEn or NE/R0 × R1)/R2

While R0 is the ratio of energy-yielding ingredients in the basal diet (96.76% and 97.30% for grower and finisher diets, respectively), R1 is the ratio of energy-yielding ingredients other than ESBM in test diets (71.76% and 72.30% for grower and finisher diets, respectively), R2 is the ratio of ESBM in test diet (25.00%).

The AME, AMEn, and NE values of ESBMs were then corrected by measured GE as follows:

Energy values (AME, AMEn or NE) of ESBM (MJ/kg) = calculated AME, AMEn or NE/calculated GE × measured GE

### 2.6. Statistical Analyses

The chamber was considered an experiment unit. A one-way analysis of variance was conducted on growth performance, nitrogen balance, energy values, and energy balance using SPSS software 24.0 (SPSS Inc., Chicago, IL, USA). The “dietary treatment” was considered as a fixed variable, while the “chamber” and “batch” were considered as random variables. The results were displayed using the main effect of dietary treatment. Duncan’s method was used to make multiple comparisons. The energy values of ESBMs were analyzed using a two-way analysis of variance. “Age of birds” and “ESBMs” were considered as main effects. In addition, the variation of ESBMs was analyzed using the principal component analysis (**PCA**) procedure with “FactoMineR” and “factoextra” packages using R 4.3.3 software. Differences were considered significant at *p* < 0.05.

## 3. Results

The nutrient contents of ESBMs are presented in Table 1. The concentrations of CP and EE in 3 ESBMs ranged from 43.46% to 46.31% and 0.80% to 0.98%, respectively (as fed basis). The percentage of GE was greater in ESBM2 (17.46 MJ/kg) compared with ESBM1 and ESBM3 (17.15 MJ/kg and 17.26 MJ/kg, respectively), while the contents of NDF and ADF were higher in ESBM1 (11.66% and 8.94%) compared with ESBM2 (11.03% and 6.50%) and ESBM3 (9.60% and 5.98%). In addition, the contents of most amino acids in the 3 ESBMs were similar. 

Effects of dietary characteristics on growth performance, nitrogen balance, energy utilization, and energy balance of broilers from 14 to 16 day and 28 to 30 day are shown in Table 4 and Table 5, respectively. BW, FI, AME intake, and AME intake/BW gain of broilers were not affected by different diets regardless of age (*p* > 0.05). Compared with basal diets, test diets increased nitrogen intake and nitrogen excreta of broilers irrespective of bird age (*p* < 0.01). As a result, a reduction of RN was observed in birds fed basal diet from 28 to 30 day (*p* < 0.05); however, the RN was not influenced by test diets from 14 to 16 day (*p* > 0.05). Compared with test diets, the AME, AMEn, and AME/GE were significantly higher in basal diet for broilers from 14 to 16 day (*p* < 0.001). Specifically, substituting ESBM1 and ESBM2 increased RE as a protein in broilers from 14 to 16 day compared with basal diet (*p* < 0.05). As expected, substituting ESBMs increased broilers’ RE as protein from 28 to 30 day (*p* < 0.05). Correspondingly, the RE as fat was significantly higher in broilers fed with basal diet from 28 to 30 day compared with those fed ESBM1 and ESBM3 diets (*p* < 0.05). Although dietary AME values were similar from 28 to 30 day (*p* > 0.05), test diets decreased AMEn values compared with basal diet (*p* < 0.05). Additionally, the NE, RQ, HP, and HI were not affected by different diets irrespective of bird ages (*p* > 0.05). 

As shown in Table 6, no treatment interaction was observed (*p* > 0.05) between ESBMs and ages for energy values and energy utilization of ESBMs. The AME, AMEn, and NE values of ESBMs for broilers from 14 to 16 day varied from 9.79 to 10.88 MJ/kg, 8.24 to 9.29 MJ/kg, and 6.61 to 7.29 MJ/kg, respectively (DM basis). The AME, AMEn, and NE values of ESBMs for broilers from 28 to 30 day varied from 11.33 to 12.84 MJ/kg, 9.88 to 11.36 MJ/kg, and 5.89 to 6.77 MJ/kg, respectively (DM basis). The AME/GE, AMEn/GE. NE/AME and NE/AMEn ranged from 0.50 to 0.66, 0.42 to 0.58, 0.51 to 0.68, and 0.59 to 0.80, respectively. Interestingly, the average AMEn of ESBMs in 28-day-old birds was significantly higher than those in 14-day-old birds (10.42 vs. 8.93 MJ/kg), while the average NE/AME of ESBMs was significantly higher in 14-day-old birds compared with those in 28-day-old birds (*p* < 0.05). Correspondingly, the AME (*p* = 0.095), AME/GE (*p* = 0.092), and AMEn/GE (*p* = 0.052) of ESBMs were numerically higher in broilers from 28 to 30 day compared with those from 14 to 16 day. As age transitioned from 14 to 28 day, the NE (*p* = 0.062) and NE/AMEn (*p* = 0.084) of ESBMs were numerically decreased.

**Figure 1:**
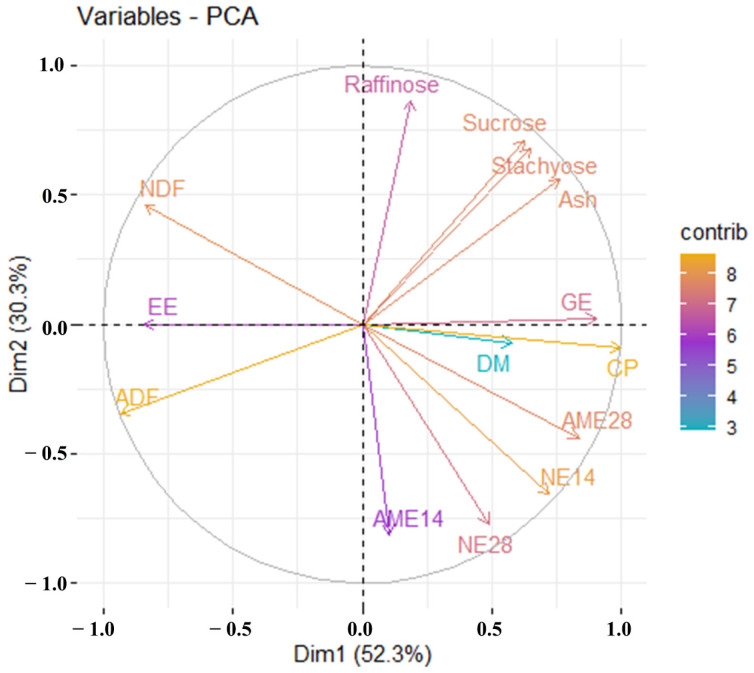
Variables of principal component analysis showing correlations between energy values and nutrient contents in ESBMs. Packages “FactoMineR” and “factoextra” in R software were used for variables of PCA analysis. An acute angle among energy values and nutrient contents indicates a positive correlation, while an obtuse angle indicates a negative correlation. Colors represent the contribution of these parameters to the variation of ESBMs (yellow, high contribution; blue, low contribution).

Variables of PCA showing correlations between energy values and nutrient contents of ESBMs (Figure 1). An acute angle among energy values and nutrient contents indicates a positive correlation, while an obtuse angle indicates a negative correlation. The energy values (AME and NE) of ESBMs in birds at different ages were positively correlated. As expected, the CP and GE contents were positively related to energy values, while the NDF content was negatively related to energy values. However, the EE content tended to be negatively correlated with energy values. There were no relationships between energy values and raffinose, sucrose, stachyose, ash, and ADF. Yellow colors represent a high parameter contribution to the variation of ESBMs, while blue colors represent a low contribution. Specifically, the contribution of dry matter content to the variation of ESBMs is relatively small, while the variation of ESBMs is highly dependent on energy values and contents of CP, GE, NDF, and ADF. 

## 4. Discussion 

### 4.1. Chemical Composition of Expanded Soybean Meal

Variations of nutrient contents among 3 ESBMs in the current study were relatively small, with GE ranging from 17.15 to 17.46 MJ/kg, NDF ranging from 9.60% to 11.66%, and crude protein ranging from 43.46% to 46.31% (as fed basis). The expanded process under high temperatures, typically near 120 °C, destroys cell structure and improves the oil extraction [28]. Therefore, the EE content of ESBMs (0.80% to 0.98%) was slightly lower than that of SBM (1.56%, as fed basis) reported by other studies [2]. Notably, the nutrient contents of ESBMs were similar to SBM except for fat content [29]. In addition, the CP and amino acid contents of ESBMs were close to values reported by Douglas and Parsons [30]. Hence, the three samples of ESBMs are regular and could be representative of measuring the energy values.

### 4.2. Effects of Diet Characteristics on Growth Performance, Nitrogen Balance, Energy Values and Energy Balance

The inclusion of ESBMs in test diets was accompanied by catabolism of protein much higher than the basal diet. Test diets with high protein contents increased nitrogen intake, excreta, and retained nitrogen. The substitution of ESBMs may correspond to a predominant deposition of protein, which differs from the normal composition of BW gain in growing animals [31]. Recently, Kim et al. [32] revealed that piglets fed high-protein diets resulted in exaggerated nitrogen and energy excretion. This may lead to an underestimation of energy values and energy utilization under the difference procedure.

Dietary characteristics, feed intake, environment temperature, and body weight could affect the HP of animals [33,34]. Including ESBMs in test diets and an unbalanced dietary crude protein resulted in a 9.76% decrease in HP in broilers (14 to 16 day). Contrary to previously published values lower than 930 kJ/kg BW^0.70^ [35,36], the HP was relatively higher in our study (1121.25 and 1008.88 kJ/kg BW^0.70^ for 14 to 16 day and 28 to 30 day, respectively). The overestimate could be explained by the variation of calorimetry chambers (close-circuit vs. open-circuit) and bird ages. In addition, the HP values were in agreement with those values (1020.00 kJ/kg BW^0.70^) obtained by Ning et al. [37]. Interestingly, grower broilers produced higher HP and HI per kg BW^0.70^ compared with finisher broilers. It is evident that the energy requirement for animals is higher in animals at an early stage than those at a later stage [38]. Moreover, finisher broilers exhibited heavier organ weights, accompanied by a more active fermentation process and lower energy efficiency [35]. It was worth noting that the HP and HI were not affected by test diets, mainly due to short-term feeding and the variation of respiratory chambers. The results were consistent with Liu et al. [2] that soybean meal substitution had no effects on HP and HI. Moreover, energy efficiencies of ME (NE/ME) were 56% and 60% for broilers from 14 to 16 day and 28 to 30 day. The results were consistent with values averaging 68% and varied by 18% in broilers [39]. 

### 4.3. Energy Values of Expanded Soybean Meals Measured by Using Difference Method

The AME values of ESBMs for broilers (ranged from 9.79 to 12.84 MJ/kg) were partly within the range (10.17 to 11.64 MJ/kg) of SBM for broilers from 14 to 42 day obtained by Khalil et al. [14]. In addition, the average AME values of soybean meal and dehulled soybean meal published by Liu et al. [2] were 10.43 and 10.80 MJ/kg. As mentioned above, Kim et al. [32] illustrated that ME concentrations of test ingredients could be underestimated if a high-protein diet is used in the difference procedure. The averaged AME/GE of ESBMs was 57.5%, slightly lower than the values (62%) of SBM for broilers published by Barzegar et al. [40] and consistent with values (56%) reported by Barzegar et al. [41]. The AME and NE values of ESBM3 were lowest among the three samples in broilers from 14 to 16 day, but they were highest from 28 to 30 day. The dietary CP of ESBM3 was the highest among the three samples; the nutrient contents account for the energy ranking of ESBMs [35]. Also, the digestive capability of broilers at different ages leads to the variation of energy values of ESBMs. Studies on NE values of SBM for broilers are relatively scanty. As illustrated by Liu et al. [2], an NE value of 6.62 MJ/kg (DM basis) for SBM (43.41% crude protein, as fed basis) was determined by using the indirect calorimetric method for 50 wk old broiler chickens. Furthermore, the NE value of 7.99 MJ/kg (DM basis) for SBM (45.8% crude protein, as-fed basis) was calculated based on chemical compositions for broilers from 21 to 35 day [42]. The reported NE value, as mentioned above, was higher than that in the current study (6.65 MJ/kg), partly due to higher fat content. Dietary fat produces less heat than protein and other nutrients during intestinal digestion and absorption, resulting in higher NE values in monogastric animals [31,40].

In addition to nutrient contents, the difference method could partially explain the differences in energy values. The difference method or regression method did not make a difference in determining the energy values of protein-rich ingredients for pigs [43]. As for the difference method, the assumption that there is no interaction between the test ingredient and other feed ingredients is obviously unfounded. Moreover, the different inclusion ratios of test ingredients may result in different energy values. When the addition level of SBM were 10%, 20%, or 30%, the measured AME values were 10.37, 10.75, or 9.85 MJ/kg (DM basis) for broilers from 9 to 12 day and 9.85, 10.33, or 10.77 MJ/kg (DM basis) for broilers from 30 to 33 day [44]. The lower inclusion ratio of ESBM in the diets necessarily means a greater degree of extrapolation and embraces results that are away from actual values [45]. Although there are drawbacks to determining energy values using the difference method, the energy values of ESBMs for broilers at different ages are still worth discussing. Further studies should be conducted to verify the energy values of ESBMs obtained in this research. 

### 4.4. Relationships between Energy Values and Nutrient Contents

The AME and NE values of ESBMs for broilers at different ages are positively correlated (Figure 1). The NE values at different ages are correlated closer than AME values; the NE values contribute greater than AME values to the variation of ESBMs, according to the angle and length of the arrows. Emerging equations were established for poultry to predict energy values using nutrient contents of diets [39,40,42] or ingredients [46,47]. In the current study, energy values are positively correlated with CP, negatively correlated with NDF, and almost independent of ADF. The results were inconsistent with previous illustrations [40], indicating that CP as a predominating nutrient exerted a positive influence on the energy values of protein-rich ingredients. In addition, the results corroborate with other studies that dietary AME value could be predicted using NDF level rather than ADF level [48,49].

### 4.5. Broiler Age Influences Energy Values of Expanded Soybean Meal

The published data on the AME values of SBM present different patterns in broilers at different ages. Batal and Parsons [50] reported that the AME value of SBM was higher in broilers at 21 days of age than that at 14 days of age. In addition, Khalil et al. [14] illustrated an increase in the AME value of SBM after 21 days and speculated that feed intake was the major factor contributing to age-dependent energy value changes. The development of microbial fermentation and improvement of nutrient digestibility could increase the energy values of diets for broilers at later growth stages [51,52]. However, another study illustrated that AME values of SBM in starter broilers (0 to 14 day) were the highest, probably due to yolk sac contribution and low endogenous losses [14]. In our study, finisher broilers tended to derive more ME but less NE per unit of ESBM than grower broilers. The following two aspects could partially explain the lower net energy of ESBMs in finisher broilers. On the one hand, 47.12% (at the grower phase) and 61.16% (at the finisher phase) of energy were used for fat deposition. As broilers grew, the carcass tended to deposit more fat and less protein [53], which decreased the efficacy of protein-rich feed [54]. On the other hand, the passage rate of feed through the intestinal tract was slower with increasing ages. The longer digesta retention time increased the fiber digestibility [55] but also promoted thermal heat generation, especially for protein feed. Therefore, birds from 14 to 16 day were more efficient in converting energy from ESBMs to body weight gain than those from 28 to 30 day. The age of birds is of considerable importance in the determination of the energy values of ingredients. 

## 5. Conclusions 

The present findings demonstrated that a bird’s age could influence energy values. Broilers at a later growth stage exhibited higher AME values of ESBMs but lower NE values. Averaged AME and NE values of ESBMs for grower broilers (14 to 16 day) and finisher broilers (28 to 30 day) were 10.48 and 6.88, 11.91 and 6.43 MJ/kg, respectively. In addition, the substitution of 25% ESBMs by using the difference method increased nitrogen intake, excreta, and energy partitioning in protein compared with basal diets. The energy values of ESBMs should be verified by using another method. Age-dependent energy values of a single ingredient, especially for protein feed, are warranted to be considered in feed formulations. 

## Figures and Tables

**Table 1:** Nutrient content and amino acid profiles of expanded soybean meals.

Item	ESBM1	ESBM2	ESBM3
Analyzed nutrient content (as fed basis)			
Dry matter (%)	88.44	88.70	88.28
Gross energy (MJ/kg)	17.15	17.46	17.26
Crude protein (%)	43.46	46.18	46.31
Either extract (%)	0.98	0.90	0.80
NDF (%)	11.66	11.03	9.60
ADF (%)	8.94	6.50	5.98
Ash (%)	6.12	6.20	6.27
Sucrose	3.47	5.25	5.61
Stachyose	1.20	3.12	3.10
Raffinose	1.09	1.93	1.74
Essential amino acid (%, DM basis)			
Argnine	2.10	2.19	2.07
Lysine	2.07	2.13	2.23
Threonine	1.51	1.53	1.56
Phenylalanine	1.58	1.71	1.48
Valine	1.83	1.96	1.95
Isoleucine	1.60	1.60	1.69
Leucine	2.84	2.94	2.97
Histidine	0.78	0.85	0.85
Nonessential amino acid (%, DM basis)			
Asparagine	4.30	4.63	4.58
Serine	2.47	2.59	2.56
Glutamate	6.28	6.54	6.51
Alanine	2.38	2.47	2.46
Proline	2.23	2.26	2.02

Abbreviations: DM = dry matter; ESBM = expanded soybean meal; NDF = neutral detergent fiber; ADF = acid detergent fiber.

**Table 2:** Ingredients composition and nutrient content of basal diets.

Items	Basal Diets	
	Grower Phase	Finisher Phase
Energy-yielding ingredients (%, as-fed basis)		
Corn	60.82	59.35
Soybean meal	21.42	20.60
Corn gluten meal	2.60	2.00
Soybean oil	4.50	6.00
DDGS	3.00	4.00
Peanut meal	3.00	4.00
L-lysine, HCl, 70%	1.00	0.96
DL-methionine, 99%	0.25	0.24
L-threonine, 99%	0.14	0.15
L-tryptophan	0.03	
Nonenergy-yielding ingredients (%, as fed basis)		
Limestone	0.90	0.92
Salt	0.27	0.26
Sodium humate	0.20	0.20
Sodium bicarbonate	0.12	0.01
Calcium bicarbonate	1.14	0.70
Choline	0.11	0.11
Vitamin-mineral premix ^1^	0.50	0.50
Total ingredients	100.00	100.00
Analyzed nutrient content (DM basis)		
Gross energy (MJ/kg)	19.52	20.07
Crude protein (%)	21.83	21.57
Either extract (%)	7.67	10.06
Ash (%)	5.88	6.10

Abbreviations: DDGS = distillers dried grains with solubles; DM = dry matter. ^1^ Vitamin-mineral premix supplied the following per kg of diet: Fe 100 mg; Cu 20 mg; Zn 100 mg; Mn 120 mg; Se 0.3 mg; I 1.0 mg; VA 10000 IU; VD_3_ 3000 IU; VE 30 mg; VK_3_ 1.5 mg; VB_1_ 2.3 mg; VB_2_ 7.8 mg; VB_6_ 5.3 mg; VB_12_ 23 mg; nicotinic acid 45 mg; pantothenic acid 12 mg; folic acid 1.0 g; biotin 5.5 mg.

**Table 3:** Dietary composition and nutrient content of test diets ^1^.

Items	Test Diets 1		Test Diets 2		Test Diets 3	
	10 to 16 Day	24 to 30 Day	10 to 16 Day	24 to 30 Day	10 to 16 Day	24 to 30 Day
Dietary composition (%, as-fed basis) ^2^						
Energy-yielding diets	71.76	72.30	71.76	72.30	71.76	72.30
Nonenergy-yielding diets	3.24	2.70	3.24	2.70	3.24	2.70
ESBM1	25.00	25.00				
ESBM2			25.00	25.00		
ESBM3					25.00	25.00
Total	100.00	100.00	100.00	100.00	100.00	100.00
Analyzed nutrient content (%, DM basis)						
Gross energy (MJ/kg)	19.47	19.70	19.41	19.79	19.46	19.82
Crude protein	28.82	28.75	29.22	28.97	29.49	29.15
Either extract	6.34	7.47	6.10	7.58	5.91	7.51
Ash	6.51	6.27	6.50	6.65	6.50	6.50

Abbreviations: ESBM = expanded soybean meal; DM = dry matter. ^1^ Broilers were fed grower diets from 10 to 16 day and fed finisher diets from 24 to 30 day. ^2^ Test diets were developed by replacing 25% of the energy-yielding ingredients, including corn, soybean meal, corn gluten meal, distillers dried grains with solubles, peanut meal and amino acids with ESBM1, ESBM2, and ESBM3.

**Table 4:** Effects of dietary characteristics on growth performance, nitrogen balance, energy values, energy utilization, and energy balance of broilers from 14 to 16 day (DM basis).

Items	Basal Diet	Test Diets			Mean	SEM	*p*-Value
		ESBM1	ESBM2	ESBM3			
Growth performance							
BW (g)	607.38	607.96	630.75	620.25	616.58	9.661	0.823
Feed intake (g/bird/day)	65.84	63.12	69.04	69.03	65.86	1.689	0.696
AME intake (kJ/bird/day) ^1^	1031.51	913.68	956.63	977.10	977.41	23.632	0.328
AME intake/BW gain (kJ/g)	16.46	14.22	13.85	14.72	14.82	0.408	0.084
Nitrogen balance (g/bird/day)							
Intake	1.77 ^b^	2.42 ^a^	2.34 ^a^	2.39 ^a^	2.23	0.066	<0.001
Excreta	0.52 ^b^	0.87 ^a^	0.83 ^a^	0.93 ^a^	0.79	0.036	<0.001
Retained ^2^	1.25	1.55	1.50	1.46	1.44	0.043	0.053
Energy values (MJ/kg)							
AME	15.68 ^a^	14.43 ^b^	14.28 ^b^	14.16 ^b^	14.66	0.169	0.001
AMEn ^3^	14.84 ^a^	13.42 ^b^	13.50 ^b^	12.97 ^b^	13.68	0.176	<0.001
NE	8.51	8.05	8.15	8.00	8.19	0.179	0.759
Energy utilization							
AME/GE	0.80 ^a^	0.74 ^b^	0.75 ^b^	0.72 ^b^	0.75	0.009	0.001
NE/AME	0.54	0.56	0.57	0.57	0.56	0.012	0.889
Energy balance (kJ/kg BW^0.70^/day)							
RE total ^4^	623.43	501.13	620.84	593.27	580.81	40.064	0.677
as protein ^4^	329.68 ^b^	403.82 ^a^	396.92 ^a^	378.88 ^ab^	377.01	10.476	0.016
as fat ^4^	293.75	97.30	223.92	214.40	203.80	28.610	0.078
RQ ^5^	0.99	0.98	0.99	0.99	0.99	0.007	0.905
HP ^6^	1201.34	1089.74	1088.36	1105.57	1123.47	21.085	0.153
HI ^7^	751.35	639.75	638.36	655.57	673.47	20.261	0.124

Abbreviations: ESBM = expanded soybean meal; BW = body weight; AME = apparent metabolizable energy; NE = net energy; RE = retained energy; RQ = respiratory quotient; HP = heat production; HI = heat increment. ^a,b^ Means within rows with different superscripts are significantly different (*p* < 0.05). ^1^ AME intake (kJ/bird/day) = apparent metabolizable energy × feed intake. ^2^ Retained nitrogen (g/bird/day) = nitrogen intake − nitrogen excreta. ^3^ AMEn (MJ/kg) = (GE intake − GE excreta − 34.41 × retained nitrogen)/feed intake. ^4^ RE (kJ/kg BW^0.70^/day) = AME intake − HP; RE as protein was calculated as RN × 6.25 × 23.86, while RE as fat was calculated by subtracting RE as protein from total RE. ^5^ RQ is the ratio of CO_2_ production to O_2_ consumption. ^6^ HP (kJ/kg BW^0.70^/day) = 16.18 × VO_2_(L/kg BW^0.70^/day) + 5.02 × VCO_2_ (L/kg BW^0.70^/day). ^7^ HI (kJ/kg BW^0.70^/day) = HP − FHP (450 kJ/kg BW^0.70^/day).

**Table 5:** Effects of dietary characteristics on growth performance, nitrogen balance, energy values, energy utilization, and energy balance of broilers from 28 to 30 day (DM basis).

Items	Basal Diet	Test Diets			Mean	SEM	*p*-Value
		ESBM1	ESBM2	ESBM3			
Growth performance							
BW (g)	1740.8	1750.6	1775.2	1737.8	1751.1	11.19	0.660
Feed intake (g/bird/day)	140.82	131.63	140.56	130.54	135.89	3.219	0.552
AME intake (MJ/bird/day) ^1^	2.11	1.83	1.96	1.82	1.93	0.050	0.127
AME intake/BW gain (kJ/g)	19.09	17.41	17.25	16.45	17.55	0.410	0.130
Nitrogen balance (g/bird/day)							
Intake	4.34 ^b^	5.74 ^a^	5.88 ^a^	5.90 ^a^	5.46	0.170	<0.001
Excreta	1.70 ^b^	2.55 ^a^	2.56 ^a^	2.56 ^a^	2.34	0.109	0.003
Retained ^2^	2.64 ^b^	3.19 ^a^	3.32 ^a^	3.35 ^a^	3.12	0.094	0.014
Energy values (MJ/kg)							
AME	14.96	13.93	13.99	14.04	14.23	0.167	0.083
AMEn ^3^	14.24 ^a^	13.05 ^b^	13.08 ^b^	13.14 ^b^	13.38	0.166	0.018
NE	9.39	8.21	8.45	8.32	8.61	0.204	0.149
Energy utilization							
AME/GE	0.75	0.71	0.71	0.71	0.72	0.008	0.227
NE/AME	0.63	0.58	0.60	0.59	0.60	0.010	0.425
Energy balance (kJ/kg BW^0.70^/day)							
RE total ^4^	671.18	459.83	530.70	468.46	532.54	39.273	0.202
as protein ^4^	315.44 ^b^	382.41 ^a^	388.92 ^a^	399.07 ^a^	371.46	11.560	0.031
as fat ^4^	355.74 ^a^	77.42 ^b^	141.78 ^ab^	69.39 ^b^	161.08	26.920	0.049
RQ ^5^	1.01	0.99	0.99	1.00	1.00	0.005	0.522
HP ^6^	1019.0	1015.2	1008.2	993.1	1008.9	14.73	0.939
HI ^7^	569.00	565.15	558.20	543.13	558.87	14.731	0.939

Abbreviations: ESBM = expanded soybean meal; BW = body weight; GE = gross energy; AME = apparent metabolizable energy; AMEn = nitrogen corrected apparent metabolizable energy; NE = net energy; RE = retained energy; RQ = respiratory quotient; HP = heat production; HI = heat increment. ^a,b^ Means within rows with different superscripts are significantly different (*p* < 0.05). ^1^ AME intake (kJ/bird/day) = apparent metabolizable energy × feed intake. ^2^ Retained nitrogen (g/bird/day) = nitrogen intake − nitrogen excreta. ^3^ AMEn (MJ/kg) = (GE intake − GE excreta − 34.41 × retained nitrogen)/feed intake. ^4^ RE (kJ/kg BW^0.70^/day) = AME intake − HP; RE as protein was calculated as RN × 6.25 × 23.86, while RE as fat was calculated by subtracting RE as protein from total RE. ^5^ RQ is the ratio of CO_2_ production to O_2_ consumption. ^6^ HP (kJ/kg BW^0.70^/day) =16.18 × VO_2_ (L/kg BW^0.70^/day) + 5.02 × VCO_2_ (L/kg BW^0.70^/day). ^7^ HI (kJ/kg BW^0.70^/day) = HP − FHP (450 kJ/kg BW^0.70^/day).

**Table 6:** Energy values and energy utilization of expanded soybean meals for broilers at different ages (DM basis) ^1^.

Treatment	Energy Values (MJ/kg) ^2^			Energy Utilization			
	AME	AMEn	NE	AME/GE	AMEn/GE	NE/AME	NE/AMEn
Age × ESBM							
ESBM1 (day 14 to 16)	10.77	9.26	6.73	0.56	0.48	0.62	0.73
ESBM2 (day 14 to 16)	10.88	9.29	7.29	0.55	0.47	0.67	0.78
ESBM3 (day 14 to 16)	9.79	8.24	6.61	0.50	0.42	0.68	0.80
ESBM1 (day 28 to 30)	11.33	9.88	6.62	0.58	0.51	0.58	0.67
ESBM2 (day 28 to 30)	11.56	10.01	5.89	0.59	0.51	0.51	0.59
ESBM3 (day 28 to 30)	12.84	11.36	6.77	0.66	0.58	0.53	0.60
Pooled SEM	0.392	0.359	0.459	0.020	0.018	0.042	0.050
Age effect							
day 14 to 16	10.48	8.93 ^b^	6.88	0.54	0.46	0.66 ^a^	0.77
day 28 to 30	11.91	10.42 ^a^	6.43	0.61	0.53	0.54 ^b^	0.62
ESBM effect							
ESBM1	11.05	9.57	6.68	0.57	0.50	0.60	0.70
ESBM2	11.22	9.65	6.59	0.57	0.49	0.59	0.69
ESBM3	11.32	9.80	6.69	0.58	0.50	0.61	0.70
*p*-value							
Age	0.095	0.046	0.062	0.092	0.052	0.022	0.084
ESBM	0.962	0.960	0.993	0.978	0.977	0.829	0.762
Age × ESBM	0.397	0.278	0.365	0.396	0.269	0.350	0.342

Abbreviations: DM = dry matter; ESBM = expanded soybean meal; AME = apparent metabolizable energy; NE = net energy; AME/GE = apparent metabolizable energy to gross energy ratio. ^a,b^ Means within rows with different superscripts are significantly different (*p* < 0.05). ^1^ Energy values of ESBMs were calculated by energy values of diets containing 25% ESBMs as follows: Calculated energy values (GE, AME, AMEn or NE) of ESBM (kcal/kg) = (test diet GE, AME AMEn or NE—basal diet GE, AME, AMEn or NE/R0 × R1)/R2. R0 are 96.76% and 97.30% for grower and finisher diets; R1 are 71.76% and 72.30% for grower and finisher diets; R2 is the ratio of ESBM in test diet (25.00%). ^2^ The AME, AMEn, and NE values of ESBMs were corrected by measured GE of ESBMs. Briefly, AME, AMEn, and NE were calculated as “calculated AME, AMEn or NE/calculated GE × measured GE”.

## Data Availability

The original contributions presented in the study are included in the article/Supplementary Material; further inquiries can be directed to the corresponding author/s.

## Supplementary Materials

The following supporting information can be downloaded at: https://www.mdpi.com/article/10.3390/ani14081198/s1, Table S1: Effects of Effects of dietary characteristics on body weight, metabolic body weight and respiratory data of broilers.
