# Supplementary material for: Broiler Age Differently Affects Apparent Metabolizable Energy and Net Energy of Expanded Soybean Meal
Source: Animals (Basel). 2024 Apr 16;14(8):1198. doi: 10.3390/ani14081198 (PMC11047715; doi:10.3390/ani14081198)
Supplement: Supplementary file 1 [file animals-14-01198-s001.zip › animals-2948096-supplementary.pdf]

**Table S1.** Effects of dietary characteristics on body weight, metabolic body weight and respiratory data of broilers.

| Items | Basal diet | Test diets | | | Mean | SEM | p-value |
|---|---|---|---|---|---|---|---|
| | | ESBM1 | ESBM2 | ESBM3 | | | |
| From 14 to 16 day | | | | | | | |
| Initial BW (g) | 417.46 | 412.79 | 411.79 | 429.46 | 417.88 | 6.878 | 0.813 |
| Final BW (g) | 607.38 | 607.96 | 630.75 | 620.25 | 616.58 | 9.661 | 0.823 |
| $BW^{0.70}$ | 626.09 | 624.15 | 633.35 | 636.49 | 630.02 | 6.458 | 0.906 |
| $O_2$ (L/kg $BW^{0.70}$/d) | 56.90 | 51.75 | 51.43 | 51.04 | 52.78 | 1.043 | 0.150 |
| $CO_2$ (L/kg $BW^{0.70}$/d) | $55.96^{a}$ | $50.34^{b}$ | $50.62^{b}$ | $49.97^{b}$ | 51.72 | 0.857 | 0.029 |
| From 28 to 30 day | | | | | | | |
| Initial BW (g) | 1410.33 | 1435.33 | 1433.25 | 1396.42 | 1418.83 | 12.250 | 0.653 |
| Final BW (g) | 1740.75 | 1750.58 | 1775.17 | 1737.83 | 1751.08 | 11.186 | 0.660 |
| $BW^{0.70}$ | 1248.99 | 1246.31 | 1275.57 | 1252.49 | 1255.84 | 10.096 | 0.750 |
| $O_2$ (L/kg $BW^{0.70}$/d) | 48.09 | 48.24 | 47.82 | 46.86 | 47.75 | 0.746 | 0.926 |
| $CO_2$ (L/kg $BW^{0.70}$/d) | 48.02 | 46.76 | 46.73 | 46.84 | 47.09 | 0.582 | 0.855 |

$BW^{0.70}$ calculated as average of initial and final body weight. Data of $O_2$ and $CO_2$ is automatically recorded at 3-min interval by chambers and calculated as daily. [a–b] Means within rows with different superscripts are significantly different ($p$ < 0.05).
